# Single molecule real-time sequencing data sets of *Hypericum perforatum* L. plantlets and cell suspension cultures

**DOI:** 10.1038/s41597-023-02878-6

**Published:** 2024-01-06

**Authors:** Rajendran K. Selvakesavan, Maria Nuc, Vladislav Kolarčik, Paweł Krajewski, Gregory Franklin

**Affiliations:** 1 Institute of Plant Genetics of the Polish Academy of Sciences, Strzeszyńska 34, 60-479 Poznań, Poland; 2 Department of Biotechnology, PSGR Krishnammal College for Women, Coimbatore, 641004 India; 3 Institute of Biology and Ecology, Faculty of Science, Pavol Jozef Šafárik University in Košice, Mánesova 23, SK-041 54 Košice, Slovakia

**Keywords:** Plant molecular biology, Secondary metabolism, Transcriptomics

## Abstract

*Hypericum* is a large genus that includes more than 500 species of pharmacological, ecological and conservation value. Although latest advances in sequencing technologies were extremely exploited for generating and assembling genomes of many living organisms, annotated whole genome sequence data is not publicly available for any of the *Hypericum* species so far. Bioavailability of secondary metabolites varies for different tissues and the data derived from different cultures will be a valuable tool for comparative studies. Here, we report the single molecule real-time sequencing (SMRT) data sets of *Hypericum perforatum* L. plantlets and cell suspension cultures for the first time. Sequencing data from cell suspension cultures yielded more than 33,000 high-quality transcripts from 20 Gb of raw data, while more than 55,000 high-quality transcripts were obtained from 35 Gb of raw data from plantlets. This dataset is a valuable tool for comparative transcriptomic analysis and will help to understand the unknown biosynthetic pathways of high medicinal value in the *Hypericum* genus.

## Background & Summary

*Hypericum perforatum* L. is an herbaceous perennial plant native to Europe, Asia, and North Africa, which is used in traditional medicine worldwide since ancient times^1,2^. Extracts of *H. perforatum* are used in the treatment of various stages of depression^3^. *H. perforatum* contains unique secondary metabolites, namely hypericin and hyperforin^4,5^, which are the two most studied extensively for their pharmacological properties^6,7^. Although the medicinal application of *H. perforatum* in the pharmaceutical industry has already reached a multi-billion dollar market, the pathways leading to the biosynthesis of bioactive metabolites are not understood adequately^8^. On the other hand, this species is considered as a toxic and invasive weed in some parts of the world^9,10^.

Comparative transcriptome analyzes using next generation sequencing techniques were utilized widely to understand biosynthetic pathways and other aspects of plant biology. Several attempts have been made to sequence and analyze the transcriptome of *H. perforatum de novo* to predict the unigenes involved in the biosynthesis of secondary metabolites^11,12^. However, genes implicated in biosynthesis pathway of hypericin and hyperforin are not characterized so far. Although whole genome data of *H. perforatum* was reported recently^13^, a complete annotated genome is not yet available in public for any of the *Hypericum* species.

The advent of single-molecule real-time sequencing (SMRT) has the potential to decipher the complex transcriptomes of plants, animals and microbes^14–19^. The advantage of SMRT technology lies in the ultra-long sequences, which in principle correspond to the full length of transcript sequences subjected to sequencing. In recent days, SMRT sequencing has been widely used to enrich the reference genome in expression studies of plant species or cultivars without a complete reference genome^20–23^. SMRT sequencing is also widely used in studies of alternative splicing, sequence repeats, long noncoding RNA, fusion genes, and gene discovery^14,20,24^. Genes involved in flavonoid and terpenoid biosynthesis were identified using SMRT sequencing in *Ginkgo biloba*^14^. Using SMRT technology, the complete sequences of unique genes involved in the synthesis of triterpene saponins were identified in *Panax ginseng*^25^. Similarly, full-length sequences of genes representing the enzymes for the berberine biosynthesis pathway in *Berberis koreana* were identified using SMRT sequencing^26^.

In the present study, we constructed a reference library for *H. perforatum* of PacBio Iso-Seq long reads using the SMRT sequencing approach. By aligning Illumina-based RNAseq data from plantlets and cell suspension cultures, we validated the library by obtaining the expression levels of several genes involved in secondary metabolism as these cultures differ greatly in the accumulation of secondary metabolites^27^. The transcriptome reference library for *H. perforatum* generated in this study will be a valuable tool as a reference library for comparative transcriptomic analyzes in *H. perforatum*

## Methods

### Plant material

In the present study, the *H. perforatum* cultivar ‘Helos’ (Richters Seeds, Ontario, Canada) was used, which is tetraploid (DNA ploidy, 2n~4x~32 chromosomes) and its reproduction mode is facultative apomixis, as revealed by flow cytometric analysis^28–30^.

**Fig. 1:**
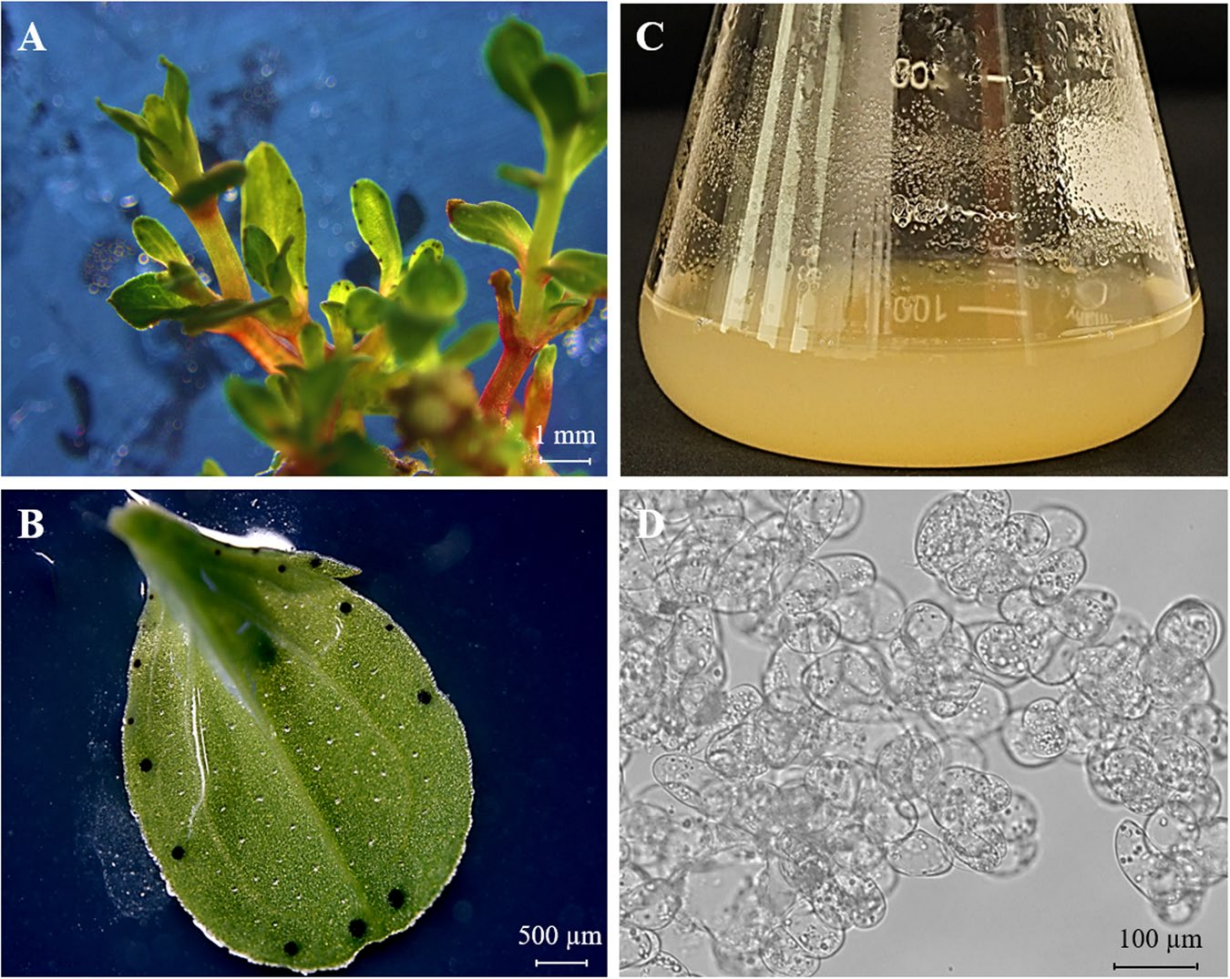
Images of *H. perforatum* plantlets (**A**), close-up of a leaf with hypericin glands (**B**), cell suspension culture (**C**), and microscopic view of cell suspension culture (**D**) used to extract RNA for Iso-Seq analysis.

Surface sterilized seeds were aseptically germinated and grown seedlings were transferred to Murashige and Skoog’s (MS - Duchefa Biochemie, Haarlem, The Netherlands) liquid medium supplemented with 0.1 mg/L α-naphthaleneacetic acid (NAA - Sigma-Aldrich, USA) and 1 mg/L 6-benzyladenine (BA - Sigma-Aldrich, USA) to establish shoot cultures containing plantlets (Fig. 1A,B). *H. perforatum* cell suspension cultures were established as reported before^1^. Cell suspension cultures were maintained in Erlenmeyer flasks (250 ml) containing MS and 0.5 mg/L NAA on an Orbi shaker (Benchmark Scientific, USA) at 110 rpm in a growth chamber with a photoperiod of 16/8 (day/night), an irradiance of 80 µmol m^−2^ s^−1^, 70% relative humidity, and a temperature of 25 °C. To keep the cells in the growth phase, 10 ml of the grown culture was subcultured into 70 ml of fresh medium once in 7 days (Fig. 1C,D).

### RNA extraction

The biomass of the cell suspension culture and plantlets was freshly harvested and ground into a fine powder in a sterile mortar and pestle with liquid nitrogen. Total RNA was isolated from 100 mg of biomass using the Sigma Spectrum RNA extraction kit (Sigma-Aldrich, USA). To remove any DNA contamination on column DNase treatment (Sigma-Aldrich, USA) was performed to purify the RNA according to the manufacturer’s instructions. RNA quantity was measured using the NanoDrop™ OneC, and RNA integrity was measured using the Agilent Bioanalyzer 2100. RNA samples with an RNA integrity number (RIN) greater than 7 were used for Illumina sequencing, and samples used for Iso-Seq library preparation had an RIN value of at least 8.

### High-throughput sequencing

Iso-Seq library preparation and sequencing were performed using the full-length PacBio cDNA libraries and sequencing kit according to the manufacturer’s protocol (Pacific Biosciences of California, Inc., Menlo Park, CA, USA) by the sequencing service provider (Novogene, Beijing, China) on the Sequel system. For RNA sequencing, the mRNA was randomly fragmented and the cDNA was synthesized using random hexamers primers. The second strand was synthesized using custom second-strand synthesis buffer (Illumina), dNTPs, RNase H, and DNA polymerase I. The double-stranded cDNA was enriched by PCR after size selection, and Illumina sequencing was performed in 150 bp paired-read mode (Novogene, Beijing, China).

### Construction of a reference library

Processing of raw sequencing data was performed by a standard Iso-seq protocol in SMRT^®^ Link v. 9.0 adhering to the guidelines provided by the manufacturer (https://www.pacb.com/wp-content/uploads/SMRT_Tools_Reference_Guide_v90.pdf), consisting of generating consensus sequences from raw data, removing primers, removing noise, clustering, polishing, and filtering high-quality transcripts. In processing, the parameter “minPasses 1” was set, and only sequences with both 5′ and 3′ adapters and a Poly(A) tail were selected to obtain the set of full-length transcripts. Annotation of transcripts was done in OmicsBox ver. 2.0.10 (https://www.biobam.com/omicsbox) by blast against nr protein database restricted to the data from taxon Magnoliopsida (e-value cut-off 1e-10) and other tools from the functional analysis suite in that software, e.g., Gene Ontology terms and KEGG pathway assignment. Completeness of the transcriptome was assessed in Busco v. 3.0 using lineage data for Eudicotyledons, with default parameters, at www.cyverse.org^31^.

### Data analysis

To validate the library, transcripts expression was assessed based on mRNA Illumina sequencing of 4 samples from cell suspension cultures and 3 samples from plantlets. Quantifiication was done using Salmon v. 0.12.0^32^ in mapping mode with the set of PacBio-based transcripts as reference sequences. Repeat sequences in transcripts were identified using RepeatMasker ver. 4.0.9 (http://www.repeatmasker.org) with RepeatMasker-RepBase^33^ Sequence Database (species maize, file RMRBSeqs.embl rel. 20181026, www.girinst.org), at public server usegalaxy.org^34^. Prediction of coding/noncoding sequences was done using the lncFinder ver. 1.1.4 package in R (ver 4.1.0) (using the “wheat” model) and PLEK^35^ (with parameter minlength = 1). Alternative splicing (AS) was investigated by gapped mapping of Illumina reads in sets of PacBio transcripts using TopHat ver. 2.1.1 (parameters: number of mismatches 1,–no-discordant), and analysis of coordinates of discovered exon junctions (corresponding to introns retained in PacBio transcripts); only AS events supported by data from all biological replications and by coverage of more than 50 Illumina reads in each replication were selected. Statistical characteristics and tests (χ2 test for comparison of frequencies, Mann-Whitney test for comparison of distributions) were computed in Genstat ver. 19^36^. Statistical graphs were made using GraphPad Prism software version 9 (GraphPad, La Jolla, CA, USA).

### Quantitative real-time PCR

To further validate the library, specific fragments of genes putatively involved in hypericin biosynthesis such as polyketide synthase1 (*PKS1*), polyketide synthase 2 (*PKS2*), isochorismate synthase 2 (*ICS2*), chorismate synthase (*CHOSYN*), polyketide cyclase (*PKC*), berberine bridge enzymes (*BBE7, BBE13, BBE15 and BBE17*)^5,9^ were analyzed with the SYBR Green-based Q-RT-PCR assay using a Lightcycler 480 real-time PCR system (Roche, Switzerland). The assay was performed as in a previous study^37^. Briefly, each 10 μL Q- RT-PCR reaction consisted of 4.2 μL diluted cDNA template (0.1 μg), 5 μL SensiFAST SYBR No- ROX (Bioline, UK), 0.4 μL forward primer (10 μM), and 0.4 μL reverse primer (10 μM). Amplification was performed under the following conditions: initial denaturation at 95 °C for 5 min, followed by 35 cycles of denaturation at 95 °C for 10 s, annealing at 60 °C for 15 s, and extension at 72 °C for 25 s. Relative expression was calculated using the 2^−ΔΔCt^ method^38^.

## Data Records

Sequence data were deposited into the functional genomics data collection (ArrayExpress) of European Bioinformatics Institute and are available with accession numbers E-MTAB-11423^39^ and E-MTAB-11325^40^. Annotation data, protein representation data and Q-RT-PCR assay data were deposited into figshare and made available to public^41^.

## Technical Validation

### Transcript characteristics

Long cDNA sequence reads of RNA isolated from cell suspension cultures and plantlets of *H. perforatum* were obtained using SMRT sequencing technology^39,42^. The length of the transcripts varied from 72 bp to 9041 bp in the cell suspension culture and 268 bp to 7986 bp in the plantlets (Table 1). In terms of a mutual blast (identity >95%), 83.19% of the transcripts from the cells and 68.21% of the transcripts from the plantlets matched. Thus, 5585 (16.81%) and 17606 (31.79%) of the transcripts were specific to cells and plantlets, respectively.

**Table 1:** Characteristics of Iso-Seq sequencing data and of obtained high-quality transcripts.

Source	Subreads bases (Gb)	Subreads number	Number of transcripts	Median length	Min length	Max length
Cell suspension culture	20.3	10 567 103	33229	2017	72	9041
Plantlets	35.5	17 767 603	55387	1991	268	7986

### Transcript annotation

From the cell suspension culture and plantlet PacBio data, 97.22 and 98.17% of the transcripts were matched to the nonredundant (nr) protein database, respectively (Table S1). Of all transcripts, 89.37% of transcripts from cell suspension culture and 90.9% of transcripts from plantlets were annotated with known proteins (Table 2). A total of 78297 unigenes from both data sets were annotated with 17254 known proteins^41,43^.

**Table 2:** Fractions of blasted transcripts annotated to protein groups.

Source	Fraction of transcripts blasted to protein groups (%)					Total number of blasted transcripts
	Named	Low quality	Uncharacterized	Hypothetical	Unnamed	
Cell suspension culture	89.37	0.02	6.70	3.75	0.17	32307
plantlets	90.90	0.01	5.91	3.08	0.11	54371
Margin	90.33	0.01	6.20	3.32	0.13	86678

Proteins were represented by 1 to 320 transcripts. Of all proteins, 3536 (20.49%) were found only in cells and 7465 (43.27%) were found only in plantlets, whereas 6253 (36.24%) proteins were observed in both data sets. The transcripts were annotated with protein sequences from 24 plant species, including *Hevea brasiliensis* (12,244 annotations), *Jatropha curcas* (10,494 annotations), *Manihot esculenta* (8422 annotations), *Ricinus communis* (7733 annotations), *H. perforatum* (1736 annotations), etc. (Table 3).

**Table 3:** Taxonomy of origin of best hits in annotation to proteins (species with number of transcripts >500).

Taxon	Number of hits in cell suspension	Number of hits in plantlets	Total
*Hevea brasiliensis*	4617	7627	12244
*Jatropha curcas*	4055	6439	10494
*Manihot esculenta*	3157	5265	8422
*Ricinus communis*	2954	4779	7733
*Populus trichocarpa*	1078	2086	3164
*Populus alba*	879	1635	2514
*Populus euphratica*	805	1500	2305
*Populus deltoides*	704	1232	1936
*Populus tomentosa*	630	1294	1924
***Hypericum perforatum***	**532**	**1204**	**1736**
*Salix suchowensis*	440	753	1193
*Pistacia vera*	388	769	1157
*Salix dunnii*	363	755	1118
*Theobroma cacao*	326	674	1000
*Tripterygium wilfordii*	338	487	825
***Hypericum monogynum***	**171**	**623**	**794**
*Salix brachista*	269	506	775
*Durio zibethinus*	267	437	704
*Corchorus olitorius*	240	381	621
*Acer yangbiense*	204	371	575
*Corchorus capsularis*	206	367	573
*Herrania umbratica*	215	357	572
*Cephalotus follicularis*	197	360	557
*Carya illinoinensis*	159	356	515

In total, the transcripts were assigned to 6103 GO terms and 1320 enzymes (Table S2). The annotations mainly related to the data sets are shown in Table S3 and Table S4. The BUSCO tool identified 48.7% and 65.2% of the genes in single copy (for the eudicotyledons) in the transcriptomes of the cell suspension cultures and plantlets, respectively (Table S2).

### Repeat Sequences in *H. perforatum* Transcript

The frequency of repeat sequences in transcripts was determined. Sequence data from cell suspension cultures contained 2.3 Mb of repeat sequences in 14619 transcripts. Similarly, plantlet data had 2.1 Mb of repeat sequences in 25729 transcripts (Table 4).

**Table 4:** Characteristics of repetitiveness in transcripts.

Source	Number of transcripts with repeats (% of all)	Total length of repeats (bp)	% of total transcriptome length
Cell suspension culture	14619 (43.99%)	2388462	3.05
Plantlets	25729 (46.45%)	2126158	1.78

**Fig. 2:**
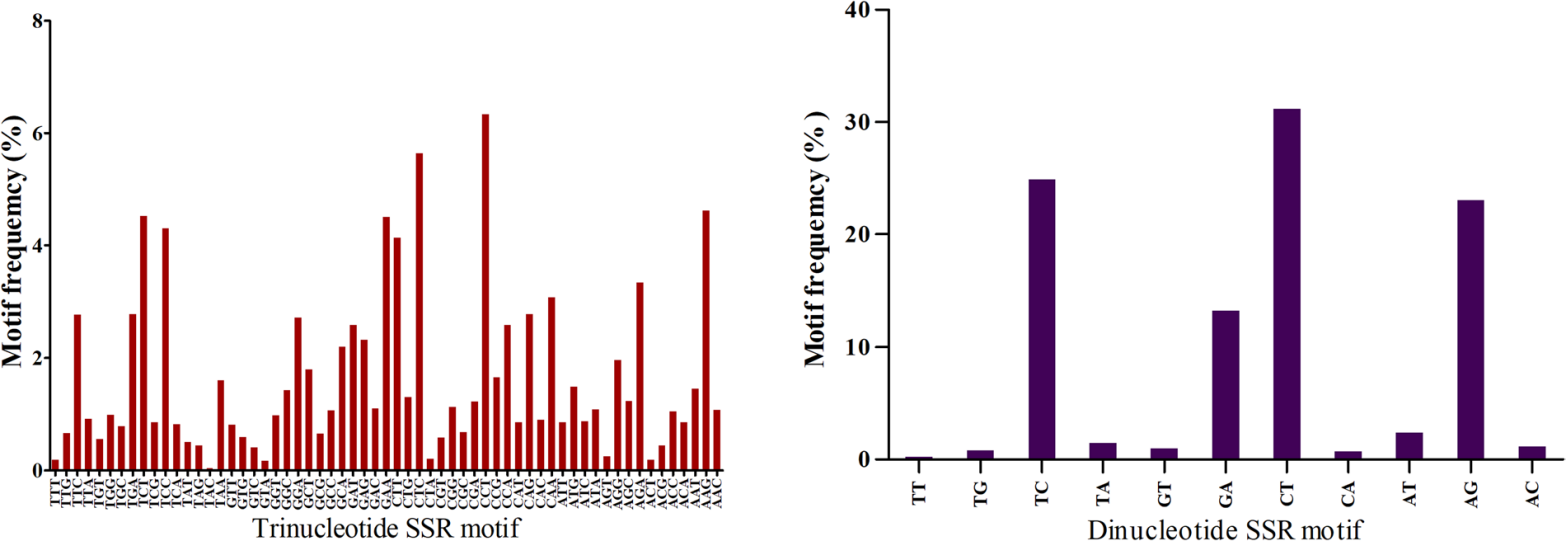
Frequency of trinucleotide and dinucleotide SSR motifs.

A total of 15833 trinucleotide sequence repeats (SSRs) and 11848 dinucleotide SSRs were identified. TCT, TCC, GAA, CTT, CTC, CCT, and AAG were the most frequently observed trinucleotide SSRs, and four simple repeats, namely TC, GA, CT, and AG, represented 92% dinucleotide SSRs (Fig. 2).

Among the identified transposon elements, long interspersed nuclear elements (LINE) and long terminal repeat (LTR) retrotransposons were predominantly represented, with frequencies higher in cell sequences (Table 5). LTR/copia and LTR/gypsy were the highly represented LTR in *H. perforatum* sequence data. Additionally, other TEs, including hAT, Mutator-like elements (MULE), P instability factor (PIF), and helitron, also were abundant in the data.

**Table 5:** Repeats with frequency significantly different from expected under the hypothesis of homogeneity between cells and plantlets (Standardized residuals obtained in the chi-square test for equality of frequencies in cell and plantlets).

Class	Frequency in cells	Frequency in plantlets	Residual in cells	Residual in plantlets
Athila0_I	21	0	6.07	−6.06
Copia-74_ALY-I	13	0	4.77	−4.77
Copia-81_ALY-I	27	0	6.88	−6.88
Copia-91_ALY-I	16	0	5.3	−5.29
Gypsy-31_ALY-I	10	0	4.19	−4.19
Gypsy-34_ALy-I	13	1	4.4	−4.4
LINE/L1	529	245	18.64	−18.64
Low_complexity	3173	6157	−5.02	5.02
LTR/Copia	222	270	4.08	−4.08
LTR/Gypsy	186	55	13.22	−13.22
rRNA	1251	1088	17.6	−17.6
Simple_repeat	16822	31425	−13.66	13.66

### Coding prediction

The selection of coding/non-coding RNAs was done by two tools: lncFinder and PLEK. Their predictions agreed for 77.08% of transcripts (70.06% as “coding”, 7.02 as “non-coding”) (Table 6).

**Table 6:** Results of prediction of coding/noncoding RNAs.

	Number of transcripts	Number of non-coding (lncFinder)	Median length of longest predicted ORF	Number of non-coding (PLEK)	Declared as non-coding by both methods
Cells	33229	5198 (15.64%)	0.974	8658 (26.06%)	3197 (9.62%)
Plantlets	55387	8226 (14.85%)	0.985	10665 (19.26%)	3020 (5.45%)
Test for difference in frequency between cells and plantlets		P = 0.001 (chi2 test)	P < 0.001 (Mann-Whitney test for location)	P < 0.001 (chi2 test)	P < 0.001 (chi2 test)

### Functional Annotation of transcripts

**Fig. 3:**
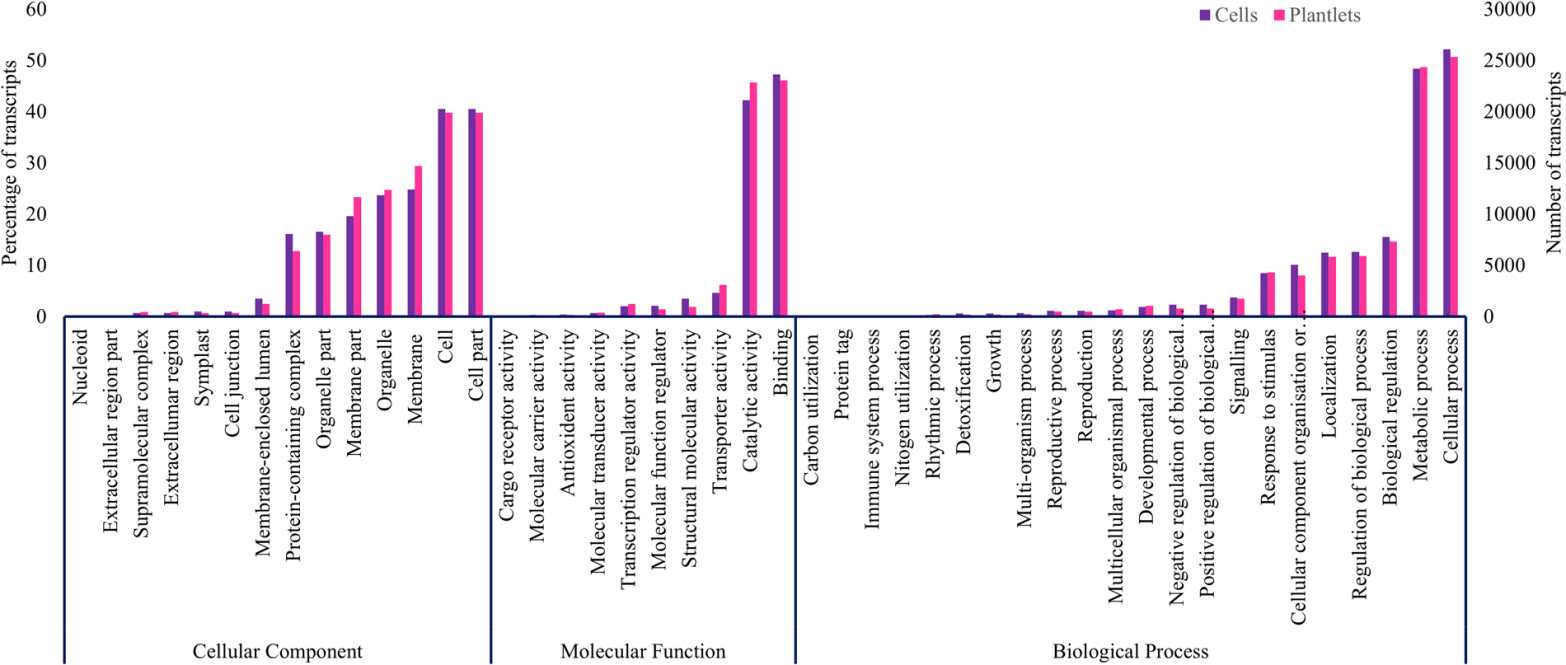
Gene Ontology classification of *H. perforatum* transcripts.

Functional classification of transcripts was carried out using Gene Ontology (GO) terms analysis. In total, 27516 transcripts from cells and 46902 transcripts from plantlets were assigned to GO terms (Fig. 3)^41,42^. Most dominant subclasses in biological process were metabolic process and cellular process.

**Fig. 4:**
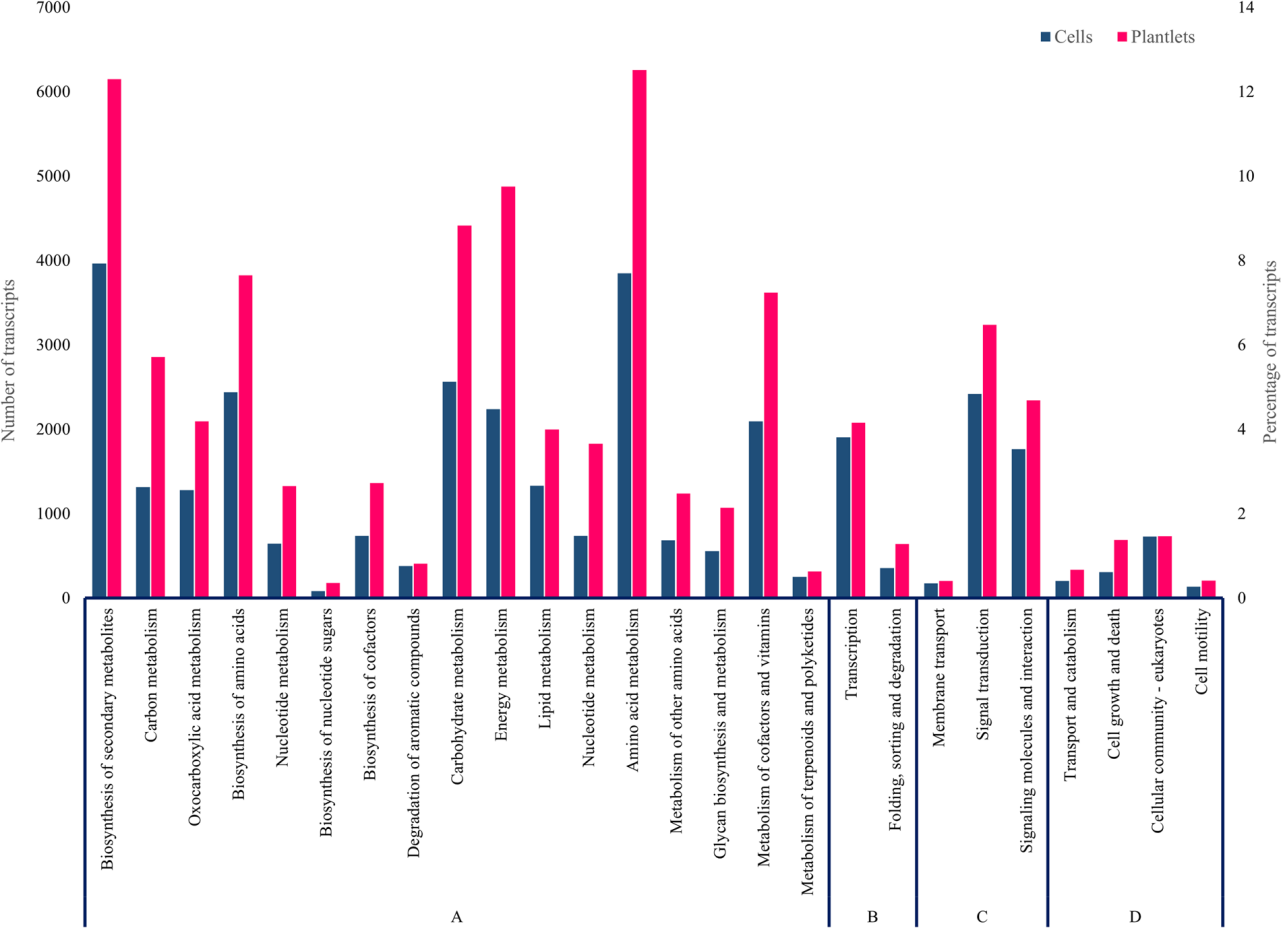
KEGG classification of *H. perforatum* transcripts. A- Metabolism; B- Genetic Information Processing; C- Environmental Information Processing; D- Cellular Processes.

In addition, Kegg’s pathway mapping was performed to identify genes involved in secondary metabolism^41,42^. Using the enzyme codes, 30900 transcripts from plantlets and 18953 transcripts from cells linked to 150 pathways were predicted after manual curation out of which, 6147 transcripts from plantlets and 3964 transcripts from cells were associated with biosynthesis of secondary metabolites (phenylpropanoid, anthocyanin, flavonoid, isoflavonoid, alkaloid, polyketide, stilbenoid, terpenoid, brassinosteroid etc) pathways (Fig. 4).

### Identification of Alternative Splicing Isoforms

**Fig. 5:**
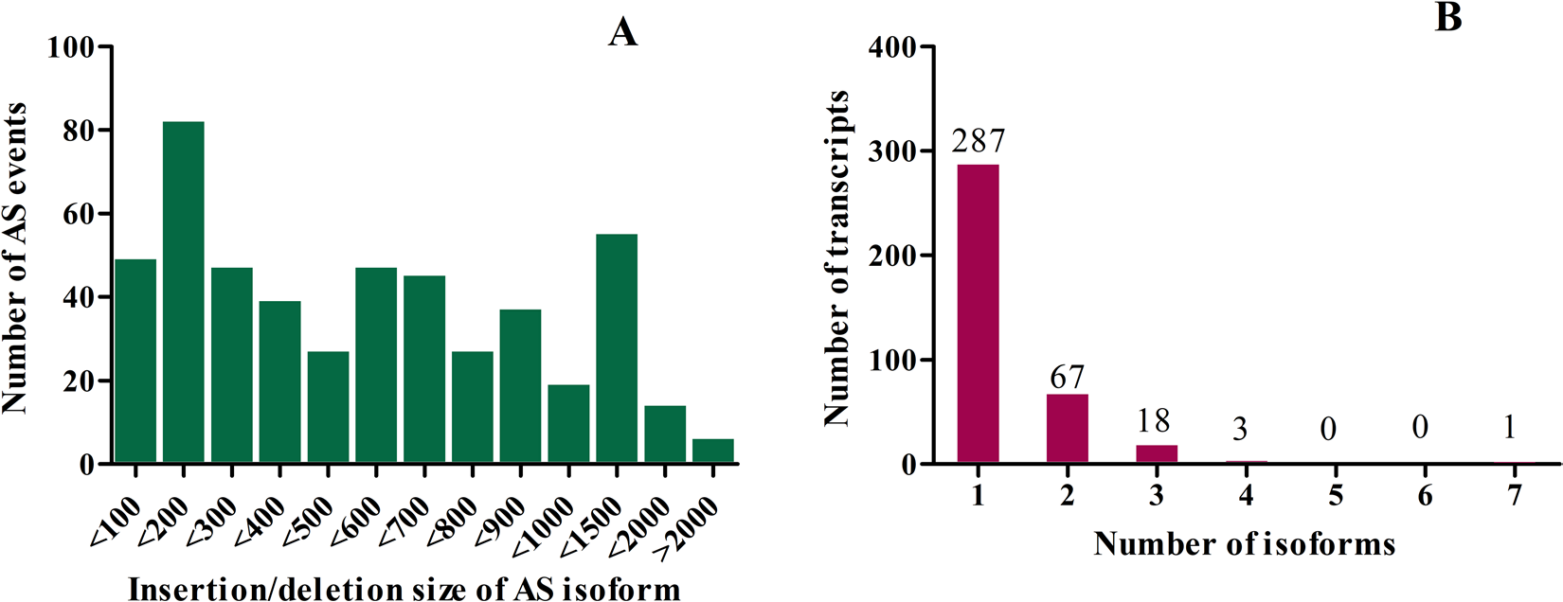
Alternative splicing in *H. perforatum* transcriptome.

In the current study, 494 alternative splicing events in 376 clusters (transcripts) were identified based on gapped mapping of Illumina reads in transcripts. In this, 130 clusters were in cell suspension culture sequence data, and 245 were in sequence data of plantlets. Insertions (intron retentions) of the size of 100–200 bp were predominantly observed (Fig. 5A). Most of the transcripts with alternative splicing events had one isoform, while a maximum of 7 isoforms was observed in one transcript (Fig. 5B).

### Expression data

Illumina sequencing of 7 samples yielded data sets with 45.0–52.4 million and 42.7–48.3 million read pairs for cells and plantlets, respectively. Expression levels (mean across all biological replicates) were calculated and transcripts were classified as “expressed” if the mean number of mapped reads was greater than 10 (Table S5).

A similar expression analysis was performed at the level of groups of transcripts assigned to the same protein, called “protein groups” (Table S5). The expression level for each protein group (in samples representing cells and plantlets) was determined as mean expression over all transcripts in the group. Protein groups were classified as “expressed” if the mean expression was greater than 10

**Fig. 6:**
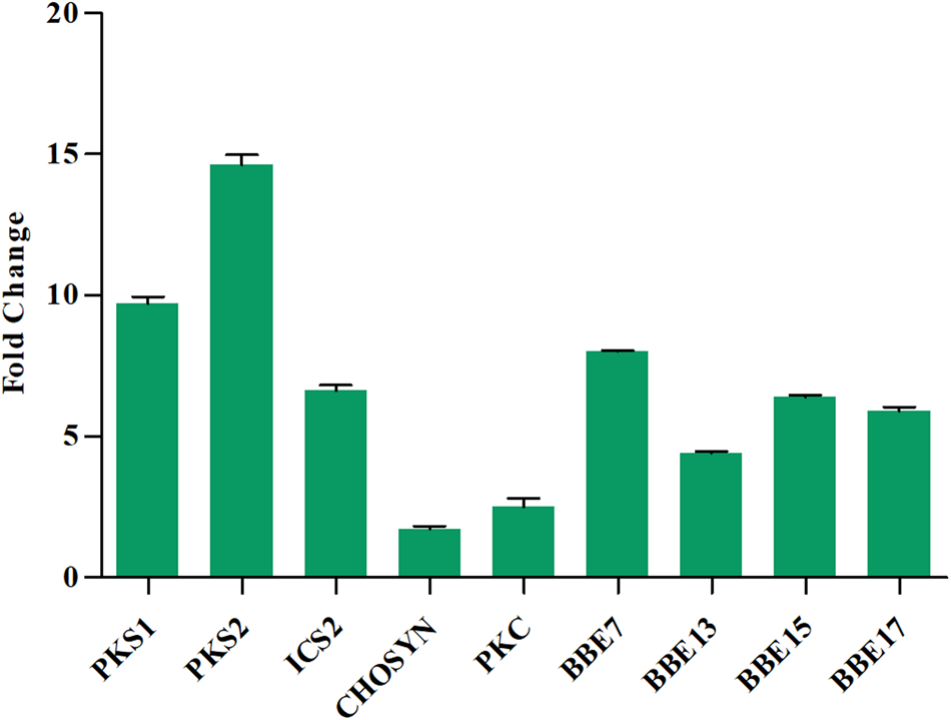
Quantitative Real-time PCR analysis of the PKS = Polyketide synthase, ICS = isochorismate synthase 2, CHOSYN = chorismate synthase, PKC = Polyketide cyclase and BBE = berberine bridge enzymes) in plantlets compared to cell suspension culture.

To confirm the quality of the expression data, 9 genes were validated for their differential expression via quantitative real-time PCR (Q-RT-PCR) analysis^44^ in plantlets and cells. (Fig. 6).

### Supplementary information

Supplementary Information

## Data Availability

All software used in this paper have been described in the Methods section with the respective version number. For this study, no custom code was generated.
