# Supplementary material for: Single molecule real-time sequencing data sets of Hypericum perforatum L. plantlets and cell suspension cultures
Source: Sci Data. 2024 Jan 6;11:42. doi: 10.1038/s41597-023-02878-6 (PMC10771467; doi:10.1038/s41597-023-02878-6)
Supplement: Supplementary file 1 — Supplementary Information [file 41597_2023_2878_MOESM1_ESM.pdf]

Supplementary Information

Single molecule real-time sequencing data sets of Hypericum perforatum L. plantlets and cell suspension culture

Rajendran K. Selvakesavan[1,2], Maria Nuc[1], Vladislav Kolarcik[3], Pawel Krajewski[1*], Gregory Franklin[1*]

[1]Institute of Plant Genetics of the Polish Academy of Sciences, Strzeszyńska 34, 60-479 Poznań, Poland

[2]Department of Biotechnology, PSGR Krishnammal College for Women, Coimbatore -641004, India

[3]Institute of Biology and Ecology, Faculty of Science, Pavol Jozef Šafárik University, Mánesova 23, SK-041 54 Košice, Slovakia

Table of Contents

Table S1 2

Table S2 2

Table S3 3

Table S4 3

Table S5 5

Table S1: Characteristics of transcript annotation in nr protein database, taxon Magnoliopsida

Source	Fraction of transcripts (%)					Total number
	Blasted, mapped, and annotated	Blasted and mapped	Blasted	Total at least blasted	Not blasted	
Cell suspension culture	75.89	7.69	13.64	97.22	2.77	33229
plantlets	77.52	7.68	12.97	98.17	1.83	55387
Margin	76.91	7.68	13.22	97.81	2.19	88616

Table S2: Completeness of transcriptomes in terms of BUSCO and overview of their annotation

Group	Completeness of transcriptome	Number of named proteins	Number of GO terms	Number of enzymes
Suspension cells	48.7%	9789	5088	1188
Plantlets	65.2%	13718	5431	1032
Margin		17254	6103	1320

Table S3: Gene ontology terms mostly specific to cell/shoot cultures

Gene ontology term	Number of transcripts in cell cultures	Number of transcripts in plantlets	Total	log2 (Fold Change)
C:nascent polypeptide-associated complex# C:GO:0005854	21	0	21	-4.20
C:photosystem II reaction center# C:GO:0009539	8	228	236	4.20
C:photosystem# C:GO:0009521	0	28	28	4.18
F:glycolate oxidase activity# F:GO:0008891	1	49	50	4.08
F:phosphoribulokinase activity# F:GO:0008974	0	26	26	4.08
F:RNA polymerase II activity# F:GO:0001055	24	0	24	-4.39
P:fructose 6-phosphate metabolic process# P:GO:0006002	0	28	28	4.18
P:fructose metabolic process# P:GO:0006000	0	28	28	4.18
P:gluconeogenesis# P:GO:0006094	0	28	28	4.18
P:negative regulation of macroautophagy# P:GO:0016242	31	1	32	-4.05
P:TORC1 signaling# P:GO:0038202	31	1	32	-4.05
P:chlorophyll biosynthetic process#P:GO:0015995	0	27	27	4.13
P:phloem development#P:GO:0010088	0	31	31	4.32
P:phototropism#P:GO:0009638	0	47	47	4.90
P:RNA export from nucleus#P:GO:0006405	21	0	21	-4.20

Table S4: Enzymes mostly specific to cell/shoot cultures

Enzyme	Number of transcripts in cell cultures	Number of transcripts in plantlets	Total	log2 (Fold Change)
15-cis-phytoene synthase# EC:2.5.1.32	0	9	9	5.98
ADP-ribose diphosphatase# EC:3.6.1.13	0	5	5	5.15
Alpha-glucosidase# EC:3.2.1.20	12	0	12	-5.05
AMP deaminase# EC:3.5.4.6	14	0	14	-5.27
Dipeptidyl-peptidase I# EC:3.4.14.1	0	5	5	5.15
Fructose-bisphosphatase# EC:3.1.3.11	1	30	31	5.82
Glutamate 5-kinase# EC:2.7.2.11	21	0	21	-5.84
Glycerate dehydrogenase# EC:1.1.1.29	0	6	6	5.41
Orotate phosphoribosyltransferase# EC:2.4.2.10	0	6	6	5.41
Protein-arginine deiminase# EC:3.5.3.15	0	9	9	5.98

Sugar-phosphatase# EC:3.1.3.23	0	15	15	6.71
1,4-dihydroxy-2-naphthoyl-CoA synthase#EC:4.1.3.36	0	5	5	5.15
2-isopropylmalate synthase#EC:2.3.3.13	17	0	17	-5.54
3,8-divinyl protochlorophyllide a 8-vinyl-reductase (NADPH)#EC:1.3.1.75	0	6	6	5.41
[Histone H3]-trimethyl-L-lysine(4) demethylase#EC:1.14.11.67	17	0	17	-5.54
Acetate--CoA ligase#EC:6.2.1.1	19	0	19	-5.70
Acting on CH or CH(2) groups#EC:1.17.7	0	5	5	5.15
Adenosine-phosphate deaminase#EC:3.5.4.17	14	0	14	-5.27
Agmatine deiminase#EC:3.5.3.12	0	9	9	5.98
Asparagine synthase (glutamine-hydrolyzing)#EC:6.3.5.4	16	0	16	-5.46
Ceramidase#EC:3.5.1.23	22	0	22	-5.91
Chlorophyll synthase#EC:2.5.1.62	0	5	5	5.15
CTP synthase (glutamine hydrolyzing)#EC:6.3.4.2	24	0	24	-6.03
Cystathionine gamma-lyase#EC:4.4.1.1	0	5	5	5.15
Cysteine--tRNA ligase#EC:6.1.1.16	12	0	12	-5.05
Endo-alpha-N-acetylgalactosaminidase#EC:3.2.1.97	12	0	12	-5.05
Forming carbon-nitrogen bonds#EC:6.3.2	60	0	60	-7.34
Glutamate-5-semialdehyde dehydrogenase#EC:1.2.1.41	21	0	21	-5.84
Glycerol-3-phosphate dehydrogenase#EC:1.1.5.3	12	0	12	-5.05
GTP cyclohydrolase I#EC:3.5.4.16	12	0	12	-5.05
Heme oxygenase (biliverdin-producing)#EC:1.14.14.18	0	6	6	5.41
Heptaprenyl diphosphate synthase#EC:2.5.1.30	0	5	5	5.15
Histidinol-phosphate transaminase#EC:2.6.1.9	0	5	5	5.15
Inositol 3-alpha-galactosyltransferase#EC:2.4.1.123	0	6	6	5.41
L-arabinokinase#EC:2.7.1.46	16	0	16	-5.46
Lanosterol synthase#EC:5.4.99.7	17	0	17	-5.54
N-(long-chain-acyl)ethanolamine deacylase#EC:3.5.1.60	13	0	13	-5.17
Ornithine carbamoyltransferase#EC:2.1.3.3	0	6	6	5.41
Orotidine-5'-phosphate decarboxylase#EC:4.1.1.23	0	6	6	5.41
Phosphoribulokinase#EC:2.7.1.19	0	26	26	7.49
Primary-amine oxidase#EC:1.4.3.21	19	0	19	-5.70
Sedoheptulose-bisphosphatase#EC:3.1.3.37	0	8	8	5.81
Tetrahydrofolate synthase#EC:6.3.2.17	16	0	16	-5.46
Urease#EC:3.5.1.5	15	0	15	-5.37

Table S5: Expression of transcripts in cell and plantlets based on Illumina data

Transcripts found in	% expressed in				Margin
	none	cells only	plantlets only	both cultures	
Cells	7.25	13.59	1.34	77.82	5585
Plantlets	14.78	2.09	44.29	38.84	17606
Both cultures	20.68	15.03	27.09	37.21	65425
Margin	18.66	12.37	28.88	40.09	88616

Protein groups

Protein groups found in	% expressed in				Margin
	none	cells only	plantlets only	both cultures	
Cells	9.30	14.93	0.99	74.77	3536
Plantlets	8.57	1.50	37.01	52.91	7465
Both cultures	0.59	1.50	2.22	95.68	6253
Margin	5.83	4.25	17.02	72.89	17254
